# Robotic versus open pancreaticoduodenectomy in elderly patients: a meta-analysis

**DOI:** 10.3389/fonc.2026.1868703

**Published:** 2026-07-14

**Authors:** Cuifang Zeng, Lin Xie, Jie Zhang, Gang Tang, Rongxing Zhou

**Affiliations:** 1 Division of Biliary Tract Surgery, Department of General Surgery, West China Hospital, Sichuan University, Chengdu, Sichuan, China; 2 Outpatient Department, West China Hospital, Sichuan University, Chengdu, Sichuan, China

**Keywords:** meta-analysis, morbidity, mortality, open pancreaticoduodenectomy, robotic pancreaticoduodenectomy

## Abstract

**Objective:**

With the aging population and increasing indications for pancreatic surgery, more elderly patients are being considered for pancreaticoduodenectomy (PD). Robotic pancreaticoduodenectomy (RPD) has been increasingly adopted; however, its safety and efficacy in elderly patients remain controversial. This systematic review and meta-analysis aimed to compare the perioperative outcomes of RPD versus open pancreaticoduodenectomy (OPD) in elderly patients.

**Methods:**

A systematic review and meta-analysis was conducted in accordance with PRISMA guidelines and registered in PROSPERO. PubMed, Cochrane Library, Scopus, EMBASE, and Web of Science were searched from inception to February 18, 2026. Studies comparing RPD and OPD in elderly patients were included. Odds ratios (ORs) and mean differences (MDs) with 95% confidence intervals (CIs) were calculated.

**Results:**

A total of 6 studies including 2, 965 patients (RPD group: 482 patients; OPD group: 2, 483 patients) were included. Compared with the OPD group, the RPD group had lower overall morbidity (OR, 0.45; 95% CI, 0.22–0.92), fewer major complications (OR, 0.68; 95% CI, 0.49–0.94), less intraoperative blood loss (MD, -119.25 mL; 95% CI, -141.36 to -97.14), lower blood transfusion rates (OR, 0.50; 95% CI, 0.34–0.72), and shorter hospital stay (MD, -1.63 days; 95% CI, -2.88 to -0.38). No significant differences were observed between the groups in mortality (OR, 0.99; 95% CI, 0.54–1.82), operative time (MD, 100.76 min; 95% CI, -57.92 to 259.43), postoperative pancreatic fistula (OR, 0.69; 95% CI, 0.48–1.00), delayed gastric emptying (OR, 1.42; 95% CI, 0.99–2.02), or reoperation rates (OR, 1.24; 95% CI, 0.72–2.13).

**Conclusions:**

This study suggests that RPD is a safe and feasible approach for elderly patients, with perioperative outcomes comparable to those of OPD. Additionally, RPD may be associated with reduced postoperative morbidity, less intraoperative blood loss, lower transfusion requirements, and shorter hospital stay. High-quality randomized controlled trials are warranted to validate these findings and further assess long-term outcomes.

**Systematic Review Registration:**

https://www.crd.york.ac.uk/PROSPERO/, identifier CRD420261444617.

## 1 Introduction

With increasing life expectancy and improvements in overall health status, the proportion of the elderly population is rising steadily worldwide (1, 2). As early as 2017, the number of older adults had more than doubled compared to 1980, and it is projected to double again by 2050 (1). This demographic shift poses significant challenges to surgical practice, particularly in complex procedures. Pancreaticoduodenectomy (PD), also known as the Whipple procedure, remains a cornerstone treatment for pancreatic cancer and periampullary diseases. However, PD is a technically demanding operation associated with substantial postoperative morbidity and mortality (3, 4). Compared with younger patients, elderly individuals require more meticulous perioperative management. This is largely attributable to the higher prevalence of comorbidities such as diabetes mellitus, hypertension, and coronary artery disease in this population (2). Consequently, elderly patients often exhibit reduced physiological reserve and are at increased risk of postoperative complications and mortality, especially following major abdominal surgery (5, 6). A study by Liu et al. (6) demonstrated that elderly patients undergoing PD experienced higher complication rates and longer postoperative hospital stays compared with their younger counterparts.

In recent years, minimally invasive surgery has been increasingly adopted in hepatopancreatobiliary procedures due to its advantages of reduced surgical trauma and faster recovery (7–9). Compared with conventional laparoscopy, robotic surgery offers enhanced dexterity, high-definition three-dimensional visualization, and improved tremor filtration. By combining the benefits of minimally invasive techniques while overcoming some limitations of laparoscopy, robotic pancreaticoduodenectomy (RPD) has emerged as a promising alternative (4, 10). Theoretically, these advantages may translate into improved perioperative outcomes in elderly patients. Several studies (11–16) have explored the efficacy of RPD in this population; however, whether RPD confers superior outcomes compared with open pancreaticoduodenectomy (OPD) in elderly patients remains controversial. Notably, there is still a lack of comprehensive systematic reviews and meta-analyses addressing this issue.

Therefore, we conducted a systematic review and meta-analysis of available evidence to compare perioperative outcomes between RPD and OPD in elderly patients. Our findings aim to provide evidence-based guidance for surgeons in selecting optimal surgical strategies for this growing and vulnerable population.

## 2 Methods

### 2.1 Search strategy

This systematic review and meta-analysis was conducted in accordance with the Preferred Reporting Items for Systematic Reviews and Meta-Analyses (PRISMA) guidelines. The study protocol was prospectively registered in the PROSPERO database. A comprehensive literature search was performed in PubMed, Cochrane Library, Scopus, EMBASE, and Web of Science from database inception to February 18, 2026. Search terms are presented in **Table 1**. Two reviewers (C.Z. and L.X.) independently conducted the literature search. To ensure completeness, the reference lists of all eligible articles and relevant reviews were manually screened for additional studies. No restrictions were imposed on publication date, language, or geographic region. Any discrepancies between reviewers were resolved through discussion, with consultation from a third reviewer when necessary.

**Table 1:** Detailed search strategies for each database.

Database	Search term (from inception to February 18, 2026)	Number
PubMed (All Fields)	((Robot*) OR (Robot-assisted) OR (Robotic-assisted) OR (Da Vinci) OR Robotic) AND (octogenarian OR older OR elder*) AND ((pancreatoduodenectomy) OR (Pancreaticoduodenectom*) OR (Duodenopancreatectom*) OR (Whipple) OR (Whipple’s procedure) OR (Kausch-Whipple) OR (Kausch-Whipple procedure))	31
Embase (All Fields)	((Robot*) OR (Robot-assisted) OR (Robotic-assisted) OR (Da Vinci) OR Robotic) AND (octogenarian OR older OR elder*) AND ((pancreatoduodenectomy) OR (Pancreaticoduodenectom*) OR (Duodenopancreatectom*) OR (Whipple) OR (Whipple’s procedure) OR (Kausch-Whipple) OR (Kausch-Whipple procedure))	104
Cochrane Library Trials (All Fields)	((Robot*) OR (Robot-assisted) OR (Robotic-assisted) OR (Da Vinci) OR Robotic) AND (octogenarian OR older OR elder*) AND ((pancreatoduodenectomy) OR (Pancreaticoduodenectom*) OR (Duodenopancreatectom*) OR (Whipple) OR (Whipple’s procedure) OR (Kausch-Whipple) OR (Kausch-Whipple procedure))	4
Web of Science (All Fields)	((Robot*) OR (Robot-assisted) OR (Robotic-assisted) OR (Da Vinci) OR Robotic) AND (octogenarian OR older OR elder*) AND ((pancreatoduodenectomy) OR (Pancreaticoduodenectom*) OR (Duodenopancreatectom*) OR (Whipple) OR (Whipple’s procedure) OR (Kausch-Whipple) OR (Kausch-Whipple procedure))	80
Scopus (Title, abstract, keywords)	((Robot*) OR (Robot-assisted) OR (Robotic-assisted) OR (Da Vinci) OR Robotic) AND (octogenarian OR older OR elder*) AND ((pancreatoduodenectomy) OR (Pancreaticoduodenectom*) OR (Duodenopancreatectom*) OR (Whipple) OR (Whipple’s procedure) OR (Kausch-Whipple) OR (Kausch-Whipple procedure))	111

### 2.2 Study selection

Studies were considered eligible if they met the following predefined inclusion criteria:

- Population: Elderly patients requiring PD. Elderly patients were defined as patients aged 70 years or more;
- Intervention: RPD;
- Comparison: OPD;
- Outcomes: primary outcomes included mortality, overall morbidity, major complications (Clavien–Dindo ≥3), and length of stay. Secondary outcomes included blood loss, operative time, reoperation, blood transfusion, delayed gastric emptying (DGE), and postoperative pancreatic fistula (POPF);
- Study design: cohort studies, and case-control studies, and randomized controlled trials (RCTs).

Exclusion criteria were as follows: animal studies, duplicate publications, reviews, editorials, conference abstracts, case reports, and single-arm studies without a comparator group.

### 2.3 Data extraction

Data extraction was independently performed by two reviewers (C.Z. and L.X.) using a standardized data collection form. Extracted variables included: first author, year of publication, country, study design, sample size, demographic characteristics (age and sex), and all relevant outcomes (mortality, overall morbidity, major complications, length of stay, intraoperative blood loss, operative time, reoperation, blood transfusion, DGE, and POPF). When data were incomplete or unclear, attempts were made to contact the corresponding authors for clarification. Any discrepancies in data extraction were resolved through discussion or consultation with a third reviewer (R.Z.).

### 2.4 Quality assessment

The methodological quality of the included studies was independently assessed by two reviewers using the Newcastle-Ottawa Scale (NOS) for observational studies. This scale evaluates studies based on three domains: selection, comparability, and outcome assessment. Studies with a NOS score greater than 6 were considered to be of high quality. Any disagreements were resolved through discussion, and if necessary, adjudicated by a third reviewer. The overall risk of bias was considered when interpreting the pooled results.

### 2.5 Statistical analysis

All statistical analyses were performed using Review Manager (RevMan, version 5.3). For continuous variables, mean differences (MDs) with corresponding 95% confidence intervals (CIs) were calculated, while odds ratios (ORs) with 95% CIs were used for dichotomous outcomes. The inconsistency index (I²) was used to quantify the degree of statistical heterogeneity. If significant heterogeneity was detected (I²> 50%), a random-effects model was applied; otherwise, a fixed-effects model was used (17). To evaluate the robustness of the pooled estimates, sensitivity analyses were conducted using a leave-one-out approach. Publication bias was assessed using funnel plots and Egger’s regression test when sufficient studies were available. Given the limited number of included studies, these analyses were interpreted with caution. A P value <0.05 was considered statistically significant.

## 3 Results

### 3.1 Study selection

**Figure 1:**
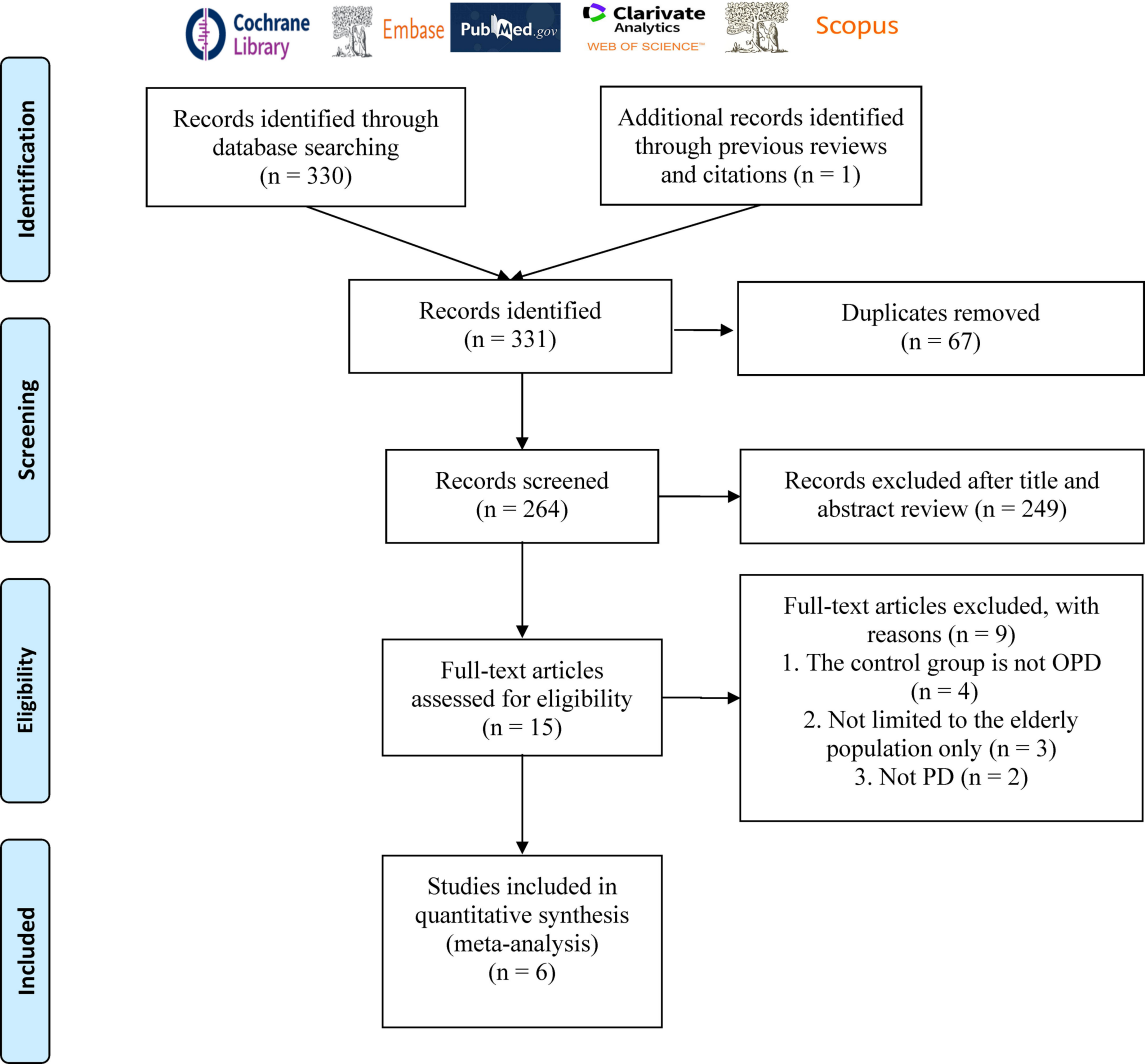
The PRISMA flowchart.

A total of 331 studies were identified from five databases, of which 67 duplicates were removed. Following title and abstract screening, 249 studies were excluded, and the full texts of the remaining 15 studies were assessed for eligibility. Ultimately, 6 studies (11–16) met the inclusion criteria and were included in the final analysis (**Figure 1**).

### 3.2 Study characteristics and quality assessment

The detailed characteristics of the included studies (11–16) are summarized in **Table 2**. These studies were conducted in various countries, including France, China, USA, and Italy, and all were retrospective cohort studies. The studies were published between 2021 and 2026, encompassing a total of 2, 965 patients (RPD group: 482; OPD group: 2483). All included studies (11–16) were considered high quality, with NOS scores greater than 6.

**Table 2:** Baseline characteristics of the included studies.

First author, year	Country	Period of study	Indication for surgery	Male	Study type	Age	Sample size	NOS
Paolini 2021	Italy	2014-2020	Malignant pancreatic or periampullary nodules	RPD: NA OPD: NA	RCS	RPD: NA OPD: NA	RPD: 29 OPD: 38	6/9
Liu 2022	China	2011-2020	Resectable benign, malignant, or borderline–malignant tumor of the pancreatic head, periampullary region, or duodenum	RPD: 86 OPD: 72	RCS	RPD: 76.9 (2.0) OPD: 76.5 (1.8)	RPD: 165 OPD: 133	7/9
Mederos 2022	USA	2015-2018	Periampullary adenocarcinoma	RPD: 62 OPD: 313	RCS, PSM	RPD: 78 (76-82) OPD: 79 (76-82)	RPD: 122 OPD: 603	8/9
Ross 2024	USA	2012-2023	Periampullary tumours	RPD:24 OPD: 19	RCS	RPD: 84 (2.7) OPD: 83 (2.6)	RPD: 42 OPD: 27	7/9
Abreu 2025	USA	2015-2021	Pancreatic adenocarcinoma	RPD: 52 OPD: 855	RCS	RPD: NA OPD: NA	RPD: 95 OPD: 1625	6/9
Wasielewski 2026	France	2019-2025	Benign or malignant disease	RPD: 14 OPD: 25	RCS	RPD: 77.64 (75.98-79.14) OPD: 77.7 (76.45-79.69)	RPD: 29 OPD: 57	8/9

NA, not available; OPD: open pancreaticoduodenectomy; PSM, propensity score matching; RCS, retrospective cohort study; RPD, robotic pancreaticoduodenectomy.

### 3.3 Meta-analysis

#### 3.3.1 Mortality

**Figure 2:**
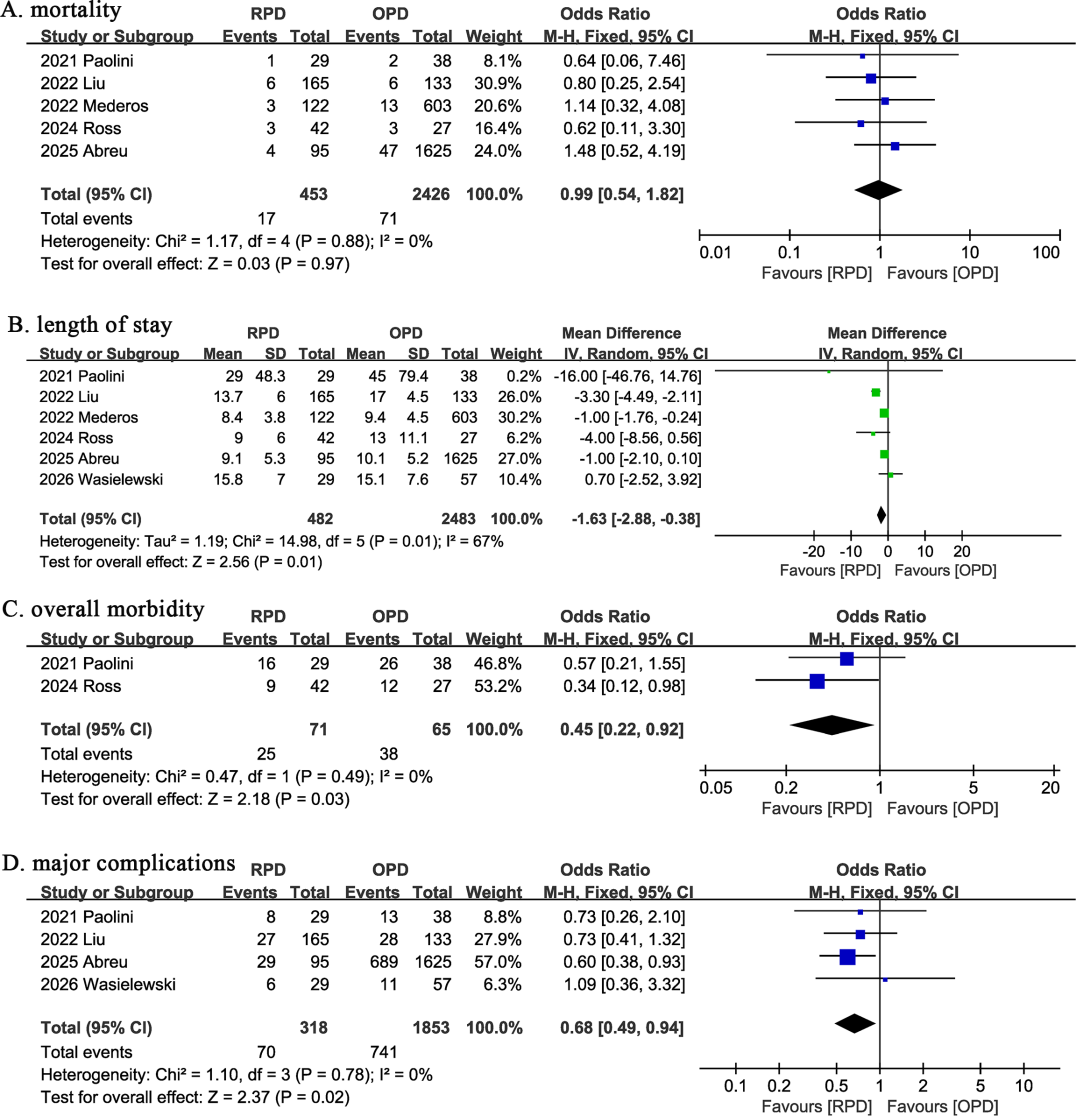
Forest plots comparing primary outcomes between the two groups. **(A)** mortality, **(B)** length of stay, **(C)** overall morbidity, and **(D)** major complications.

Five studies (11–15) assessed mortality. The combined results of the 5 studies showed that there was no significant difference between the RPD group and the OPD group regarding this outcome with low heterogeneity (OR 0.99, 95% CI 0.54, 1.82; Heterogeneity: I^2^ = 0%, P = 0.88) (**Figure 2A****; ****Table 3****).**

**Table 3:** Summary of pooled outcomes comparing robotic and open pancreaticoduodenectomy.

Outcomes	No. of studies	Events for RPD	Events for OPD	Effect size	95%CI	P	I2 (%)
Overall complications	2	25/71	38/65	0.45	0.22, 0.92	0.03	0
Mortality	5	17/453	71/2426	0.99	0.54, 1.82	0.97	0
Major complications	4	70/318	741/1853	0.68	0.49, 0.94	0.02	0
Blood transfusion	5	40/440	496/2456	0.50	0.34, 0.72	0.0002	0
POPF	4	45/411	346/2399	0.69	0.48, 1.00	0.05	0
Reoperation	4	21/318	111/1853	1.24	0.72, 2.13	0.44	0
DGE	4	63/345	141/831	1.42	0.99, 2.02	0.05	25
Blood loss	2	–	–	-119.25	-141.36, -97.14	< 0.00001	39
Operative time	3	–	–	100.76	-57.92, 259.43	0.21	100
Hospital stay	6	–	–	-1.63	-2.88, -0.38	0.01	67

“Events” applies only to dichotomous outcomes applies. For continuous variables, the pooled effect estimates were calculated using mean values and standard deviations rather than event counts.

#### 3.3.2 Length of stay

The length of the hospital stay was reported in 6 studies (11–16) According to the results of this meta-analysis, RPD significantly reduced the length of hospital stay (MD, -1.63 days; 95% CI, -2.88, -0.38, P = 0.01) (**Figure 2B****).**

#### 3.3.3 Morbidity

Two studies (11, 14) reported data on overall complication. The pooled results suggested that RPD significantly reduced the overall complication rate (OR 0.45, 95% CI 0.22, 0.92, P = 0.03), with low heterogeneity (I^2^ = 0%, P = 0.49) (**Figure 2C**). Pooled data from four studies (11, 12, 15, 16) demonstrated that, compared with OPD, RPD was associated with a significantly lower incidence of major complications (OR 0.68, 95% CI 0.49, 0.94; Heterogeneity: I^2^ = 0%, P = 0.78) (**Figure 2D**).

#### 3.3.4 Blood loss

**Figure 3:**
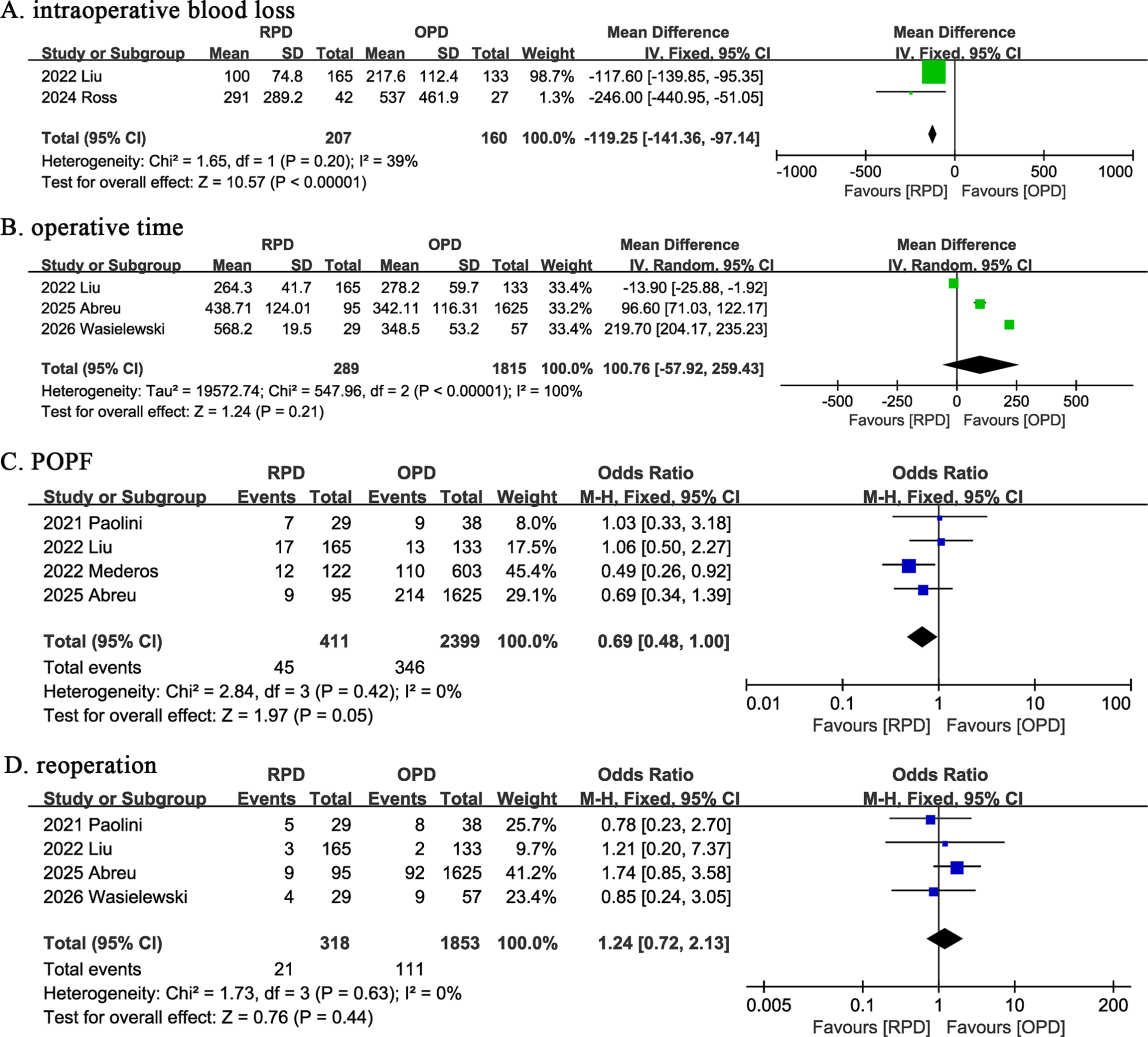
Forest plots comparing secondary outcomes between the two groups. **(A)** intraoperative blood loss, **(B)** operative time, **(C)** POPF, and **(D)** reoperation.

The intraoperative blood loss was reported in 2 studies (12, 14). The pooled analysis showed that the RPD group had significantly less intraoperative blood loss than the OPD group (MD, -119.25 mL; 95% CI, -141.36, -97.14, P < 0.00001) (**Figure 3A**).

#### 3.3.5 Operative time

Three studies (12, 15, 16) provided information on operative time. The combined results showed that the operative time was similar between the RPD group and the OPD group (MD, 100.76 min; 95% CI, -57.92, 259.43, P = 0.21; I^2^ = 100%) (**Figure 3B**).

#### 3.3.6 POPF

POPF was reported in 4 studies (11–13, 15), and the combined effect size suggested that the POPF rates were comparable between the two groups (OR 0.69, 95% CI 0.48, 1.00, P = 0.05; I^2^ = 0%) (**Figure 3C**).

#### 3.3.7 Reoperation

Four studies (11, 12, 15, 16) provided information on reoperation rates. There was no significant difference in reoperation rates (OR 1.24, 95% CI 0.72, 2.13, P = 0.44; I^2^ = 0%) (**Figure 3D**).

#### 3.3.8 DGE

**Figure 4:**
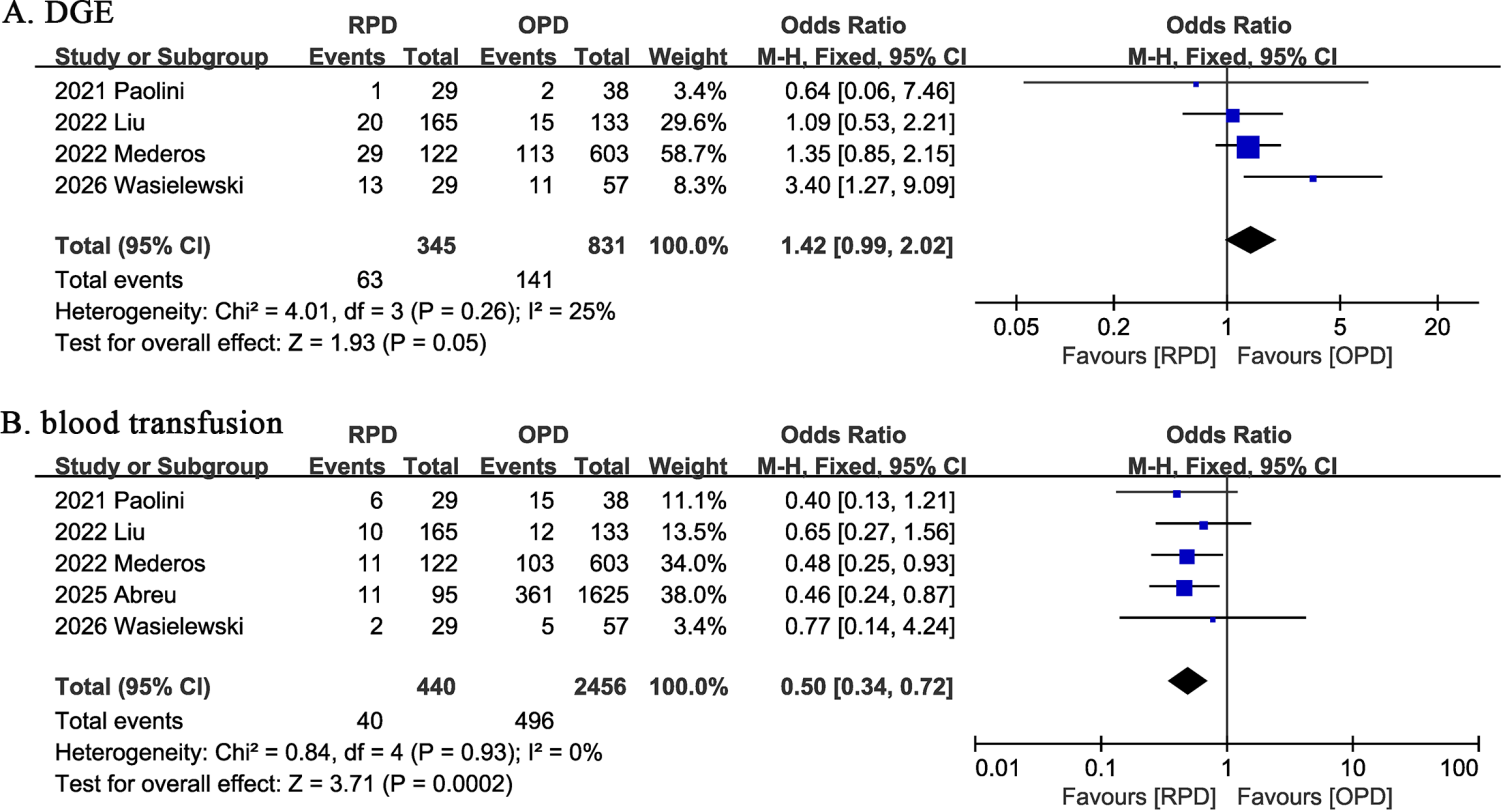
Forest plots comparing secondary outcomes between the two groups. **(A)** DGE, and **(B)** blood transfusion.

Four trials (11–13, 16) reported on DGE rates. There was no statistically significant difference between the RPD and OPD groups; however, a trend toward increased DGE in the RPD group was observed (OR 1.42, 95% CI 0.99, 2.02, P = 0.05; I^2^ = 25%) (**Figure 4A**).

#### 3.3.9 Blood transfusion

Five studies (11–13, 15, 16) compared blood transfusion rates between the RPD and OPD groups. The pooled analysis demonstrated that blood transfusion rates were significantly lower in the RPD group than in the OPD group (OR 0.50, 95% CI 0.34, 0.72, P = 0.0002) (**Figure 4B**).

### 3.4 Publication bias

**Figure 5:**
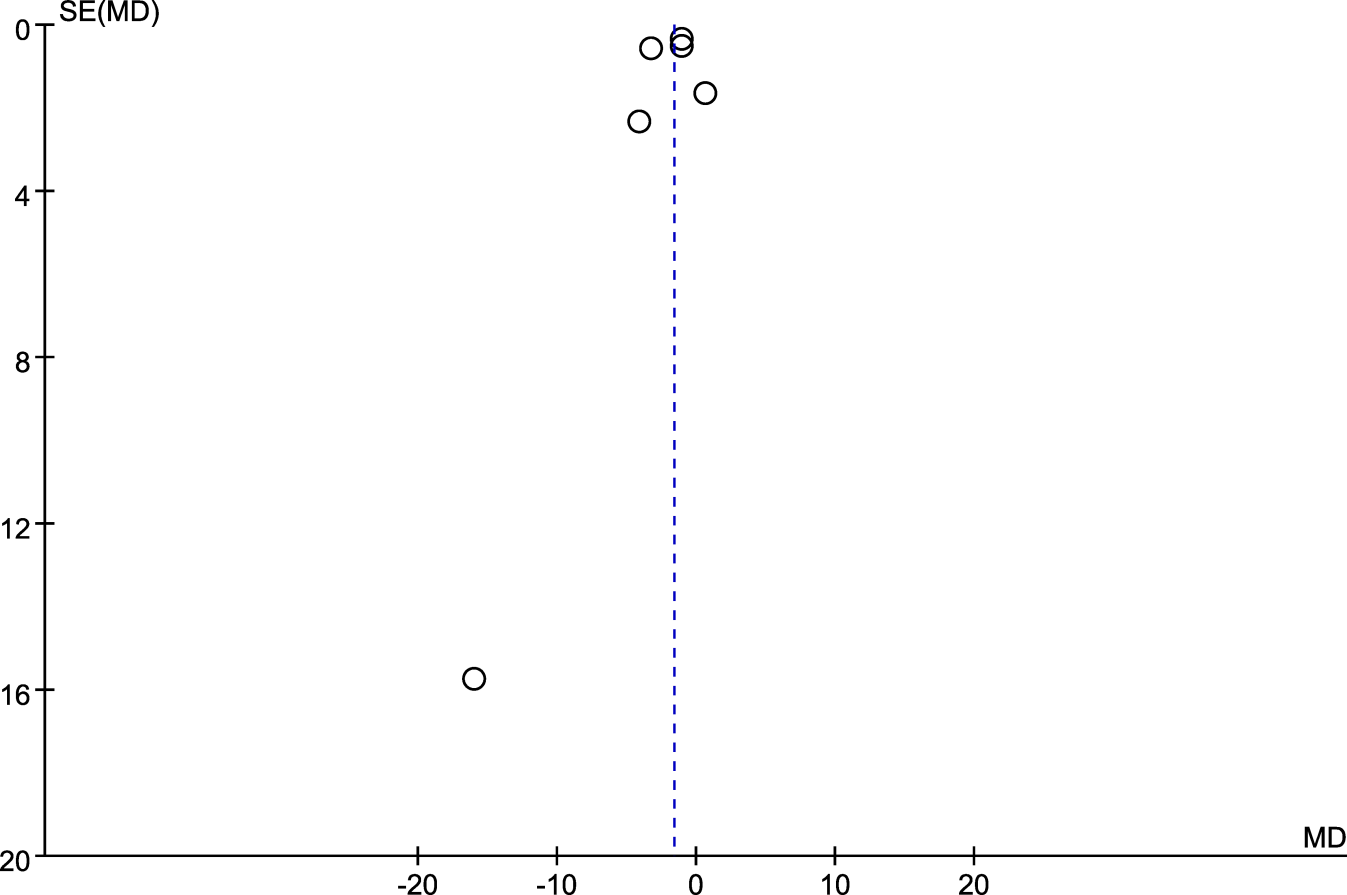
Funnel plots assessing publication bias for length of stay.

Given the limited number of included studies, publication bias was assessed only for length of hospital stay. The funnel plot and Egger’s test (P = 0.285) did not indicate significant evidence of publication bias (**Figure 5**).

### 3.5 Sensitivity analysis

Sensitivity analysis showed that no single study affected the overall effect size of the mortality, length of stay, operative time, reoperation, blood transfusion. The sensitivity analysis suggested that the total effect size of major complications changed significantly when the study by Abreu et al. (15) (OR 0.79, 95% CI 0.49, 1.25; I^2^ = 0%, P = 0.82) was excluded. The sensitivity analysis suggested that the total effect size of DGE changed significantly when the study by Paolini et al. (11) (OR 1.45, 95% CI 1.01, 2.07; I^2^ = 44%, P = 0.17) was excluded. The sensitivity analysis suggested that the total effect size of POPF changed significantly when the study by Paolini et al. (11) (OR 0.66, 95% CI 0.45, 0.98; I^2^ = 16%, P = 0.04) or Liu et al. (12) (OR 0.61, 95% CI 0.40, 0.94; I^2^ = 0%, P = 0.50) was excluded. Regarding heterogeneity, no single study substantially affected the heterogeneity of mortality, major complications, DGE, operative time, reoperation, or blood transfusion. In contrast, exclusion of the study by Liu et al. (12) markedly reduced the heterogeneity for length of hospital stay (MD, -1.00 days; 95% CI, -1.61, -0.39; I^2^ = 0%, P = 0.001).

## 4 Discussion

With the increasing adoption of robotic platforms in pancreatic surgery, RPD has emerged as an important alternative to conventional OPD. However, its safety and efficacy in elderly patients remain debated. To our knowledge, this is the first meta-analysis specifically comparing RPD and OPD in an elderly population. Based on six studies encompassing 2, 965 patients, our pooled results demonstrate that, compared with OPD, RPD is associated with significantly lower rates of overall postoperative complications and major complications, reduced intraoperative blood loss, lower transfusion requirements, and shorter length of hospital stay. No significant differences were observed between the two approaches in operative time, postoperative pancreatic fistula (POPF), or perioperative mortality. Notably, the result for DGE was of borderline statistical significance. Given the relatively wide confidence interval, this finding should be interpreted with caution, and the potential difference between RPD and OPD regarding DGE remains inconclusive. Further high-quality studies with larger sample sizes are warranted to clarify this issue. Collectively, these findings suggest that, in appropriately selected elderly patients, RPD is not only safe and feasible but may also confer advantages in perioperative recovery.

Previous comparisons of RPD and OPD have largely focused on mixed-age populations. Several retrospective studies and meta-analyses have reported that RPD is at least non-inferior to OPD in terms of safety and short-term outcomes, with some suggesting benefits in blood loss, length of stay, and major complications (18–20). Our results extend this evidence base by focusing on a high-risk subgroup and demonstrating a consistent reduction in postoperative morbidity among elderly patients. This is clinically meaningful, as older individuals typically present with a higher burden of comorbidities, diminished physiological reserve, and increased operative risk (6). Postoperative complications in this population are closely linked to prolonged recovery, higher resource utilization, and potentially worse long-term outcomes (21). Reported morbidity rates following PD in elderly patients can be as high as 50%, and the likelihood of intensive care utilization is greater than in younger cohorts (1). Therefore, the observed reduction in complications with RPD may translate into downstream benefits, although this requires confirmation in studies with long-term follow-up.

The mechanisms underlying the reduction in postoperative morbidity with RPD are likely multifactorial. First, the robotic platform provides high-definition three-dimensional visualization and articulated instruments with enhanced dexterity, enabling more precise dissection and hemostasis (10). A recent study by Tang et al. (10) demonstrated that, compared with open surgery, robotic surgery significantly reduced intraoperative blood loss and transfusion rates. These findings are consistent with our observations of decreased intraoperative blood loss and a lower transfusion rate. Second, the minimally invasive approach may attenuate the surgical stress response, thereby reducing systemic inflammation and facilitating recovery (22). Third, improved visualization and instrument control may allow for more meticulous vascular handling and anastomotic construction, potentially lowering the risk of complications such as hemorrhage and infection (23). Notably, perioperative transfusion has been associated with adverse outcomes, including increased infection rates and mortality (24); thus, the reduced transfusion requirement observed with RPD may partially mediate the overall improvement in morbidity.

Length of hospital stay is an important surrogate of postoperative recovery and is associated with overall prognosis (25, 26). Several studies in major abdominal surgery (e.g., colorectal, gastric, and hepatic resections) have shown that minimally invasive approaches can shorten hospitalization (23, 27, 28). In line with this, our analysis indicates that RPD significantly reduces length of stay in elderly patients, consistent with enhanced recovery paradigms (29). However, the interpretation of this finding requires caution. Length of hospital stay is influenced by multiple factors beyond the surgical approach itself, including the occurrence of postoperative complications, institutional discharge policies, and perioperative care protocols. Therefore, the observed reduction in hospital stay may be partially attributable to the lower rates of postoperative morbidity observed in the RPD group. In addition, variations in perioperative management among institutions may have contributed to the heterogeneity of this outcome. Future prospective studies and randomized controlled trials are needed to more accurately determine the independent impact of RPD on postoperative hospitalization in elderly patients. The interpretation of operative time similarly warrants caution because substantial heterogeneity was observed across the included studies. Several factors may explain this variability. First, RPD is characterized by a well-recognized learning curve, with operative efficiency improving considerably as surgical experience accumulates. Second, differences in institutional case volume, surgeon expertise, and the maturity of robotic surgery programs may substantially influence operative duration. Third, patient selection criteria and case complexity—including tumor burden, anatomical considerations, and the need for vascular resection—may vary across centers. Finally, differences in robotic platforms, operative techniques, and perioperative workflows may further contribute to interstudy heterogeneity. Consequently, although no statistically significant difference in operative time was identified between RPD and OPD, this finding should be interpreted cautiously and should not be regarded as definitive evidence of equivalent procedural efficiency.

This study has several strengths. We performed a comprehensive literature search across multiple major databases (PubMed, Cochrane Library, Scopus, EMBASE, and Web of Science), enhancing the completeness of evidence capture. In addition, by focusing exclusively on elderly patients, we reduced clinical heterogeneity and addressed a population that is underrepresented in the existing literature.

The current study has the following limitations. First, all included studies were retrospective, introducing an inherent risk of selection bias. Patients undergoing RPD are often treated in high-volume centers with substantial robotic experience and may be more carefully selected than those undergoing OPD. In routine clinical practice, surgeons may preferentially offer robotic surgery to physiologically fitter elderly patients with lower frailty burden, fewer comorbidities, and more favorable anatomical characteristics, particularly during the early adoption phase of RPD. Such preferential selection may have contributed to the observed reductions in postoperative complications and length of hospital stay. Although several studies applied propensity score matching or multivariable adjustment, residual confounding cannot be completely excluded. Second, substantial heterogeneity was observed for several outcomes. One important source of heterogeneity is the well-recognized learning curve associated with RPD. Increasing surgical experience has been shown to improve operative efficiency and perioperative outcomes, including complication rates, conversion rates, and operative time. Consequently, differences in institutional experience, surgeon proficiency, and case volume across studies may have significantly influenced the pooled results. This issue may be particularly relevant in elderly patients. During the early implementation phase of robotic programs, surgeons frequently adopt a cautious patient selection strategy, preferentially offering RPD to patients with favorable anatomy, lower operative risk, and fewer comorbidities. Such selection practices may contribute to improved perioperative outcomes and should be considered when interpreting comparative studies. Therefore, the favorable outcomes observed in the present analysis may be most applicable to experienced centers with established robotic programs and well-defined patient selection protocols. Furthermore, the definition of elderly patients varied slightly among the included studies. While most studies used an age threshold of 75 years, others specifically focused on octogenarians. This variation may introduce additional clinical heterogeneity, as physiological reserve, frailty status, and operative risk can differ substantially between patients aged 75 years and those aged 80 years or older. Because of insufficient reporting in the original studies, age-stratified subgroup analyses were not feasible. Third, several included studies had relatively small sample sizes, which may have increased the risk of statistical imprecision and selection bias. In addition, important geriatric variables—including frailty status, nutritional condition, functional capacity, and comorbidity burden—were inconsistently reported and rarely incorporated into adjusted analyses. The absence of these data limited the ability to perform risk-stratified analyses and may have confounded the observed associations. Future studies should incorporate standardized geriatric assessments to better characterize elderly surgical candidates. Fourth, although our analysis demonstrated potential advantages of RPD in short-term perioperative outcomes, the lack of long-term follow-up data precluded evaluation of oncologic outcomes, recurrence patterns, and overall survival. Given the established relationship between postoperative complications and long-term prognosis, particularly in patients undergoing pancreatic cancer surgery, extended follow-up is necessary to determine whether the short-term benefits of RPD translate into meaningful long-term clinical advantages. Finally, the economic impact of robotic surgery remains an important consideration. However, cost-effectiveness data were not available in the included studies, preventing meaningful assessment of the balance between clinical benefits and healthcare expenditures. Future investigations should evaluate both the clinical and economic implications of RPD in elderly populations to better inform surgical decision-making and resource allocation.

In conclusion, our meta-analysis suggests that, in elderly patients, RPD provides short-term outcomes comparable to OPD while offering potential advantages, including reduced postoperative morbidity, lower blood loss and transfusion requirements, and shorter hospital stay. When performed in experienced centers with careful patient selection, RPD may represent a feasible and potentially advantageous surgical strategy for elderly patients. Nevertheless, given the limitations of the current evidence, high-quality prospective RCTs are required to validate these findings and to further clarify the long-term and cost-effectiveness profiles of RPD in this population.

## Data Availability

The original contributions presented in the study are included in the article/supplementary material. Further inquiries can be directed to the corresponding author.
